# Successful treatment of a ruptured aneurysm at the vertebral artery-posterior inferior cerebellar artery junction and simultaneous treatment of the stenotic vertebral artery with a single flow-diverting stent: a case report

**DOI:** 10.1186/1752-1947-8-172

**Published:** 2014-05-30

**Authors:** Ljubisa Borota, Gyula Gál, Per Jonasson, Per-Åke Ridderheim

**Affiliations:** 1 Department of Radiology, Oncology and Radiation Science, Uppsala University, Uppsala, Sweden; 2 Department of Radiology, Odense University Hospital, Odense, Denmark; 3 Section of Neuroradiology, Department of Radiology, Umeå University Hospital, Umeå, Sweden; 4 Department of Neurosurgery, Umeå University Hospital, Umeå, Sweden

**Keywords:** Cerebral aneurysm, Flow-diverting stent, Parent artery, Rupture, Stenosis

## Abstract

**Introduction:**

This is the first report on the simultaneous successful treatment of a large ruptured saccular aneurysm and stenotic parent artery with a single flow-diverting stent.

**Case presentation:**

We report the case of a 68-year-old Caucasian man with occlusion of the right vertebral artery and a ruptured aneurysm at the junction of the left posterior inferior cerebellar artery-left vertebral artery that was successfully treated by the deployment of a single flow-diverting stent in the stenotic left vertebral artery. Stent deployment was complicated by thrombotic occlusion of the basilar artery, which was successfully reopened. The patient recovered completely, and follow-up angiography at 4 months and 1 year showed patent vertebral artery with gradual shrinkage of the aneurysm.

**Conclusions:**

This report contributes to the literature on treatment of large ruptured aneurysms localized in stenotic arteries and in areas of the endocranium where a mass of embolic material in the aneurysm (coils) might compromise the circulation in the parent blood vessel or compress vital brain structures.

## Introduction

Aneurysms of the posterior inferior cerebellar artery (PICA) present a therapeutic challenge because of their proximity to cranial nerves and the narrow space between the brainstem and the walls of the posterior fossa [1,2]. Other considerations, such as brainstem compression, imprinting of the aneurysm in the brainstem, an unclear relationship between the aneurysm and the parent vessel, as well as arteriosclerotic changes of the parent vessel may also reduce the range of therapeutic options. The main aims when treating a ruptured aneurysm are occlusion of the bleeding point and exclusion of the lumen of the aneurysm from the circulation. The secondary aim is to reduce the compression the aneurysm exerts on neighboring structures. Coil occlusion of the aneurysm can lead to regression of symptoms caused by the water-hammer effect [3]; however, symptoms related to compression may sometimes persist if circulating blood in the aneurysm is simply replaced by coil mesh of the same volume [4], with potentially serious consequences in cases where the aneurysm presses on or is imprinted on vital structures such as the pons or medulla [4]. Stenotic changes of the parent vertebral artery (VA) may increase the risk of thromboembolic complications during surgical or endovascular treatment of such aneurysms.

The new generation of flow-diverting stents may allow substantial change in the therapeutic approach to unruptured, broad-based intracranial aneurysms. Preliminary results based on a nonrandomized series of patients indicate that treatment of such aneurysms with flow-diverting stents can be a promising therapeutic alternative [5,6]. Ruptured aneurysms that compress vital structures, and that cannot be clipped or coiled because of treatment-related risks, represent a real therapeutic challenge. In such cases, deployment of a flow-diverting stent in the parent vessel could be the only acceptable therapeutic alternative.

## Case presentation

**Figure 1:**
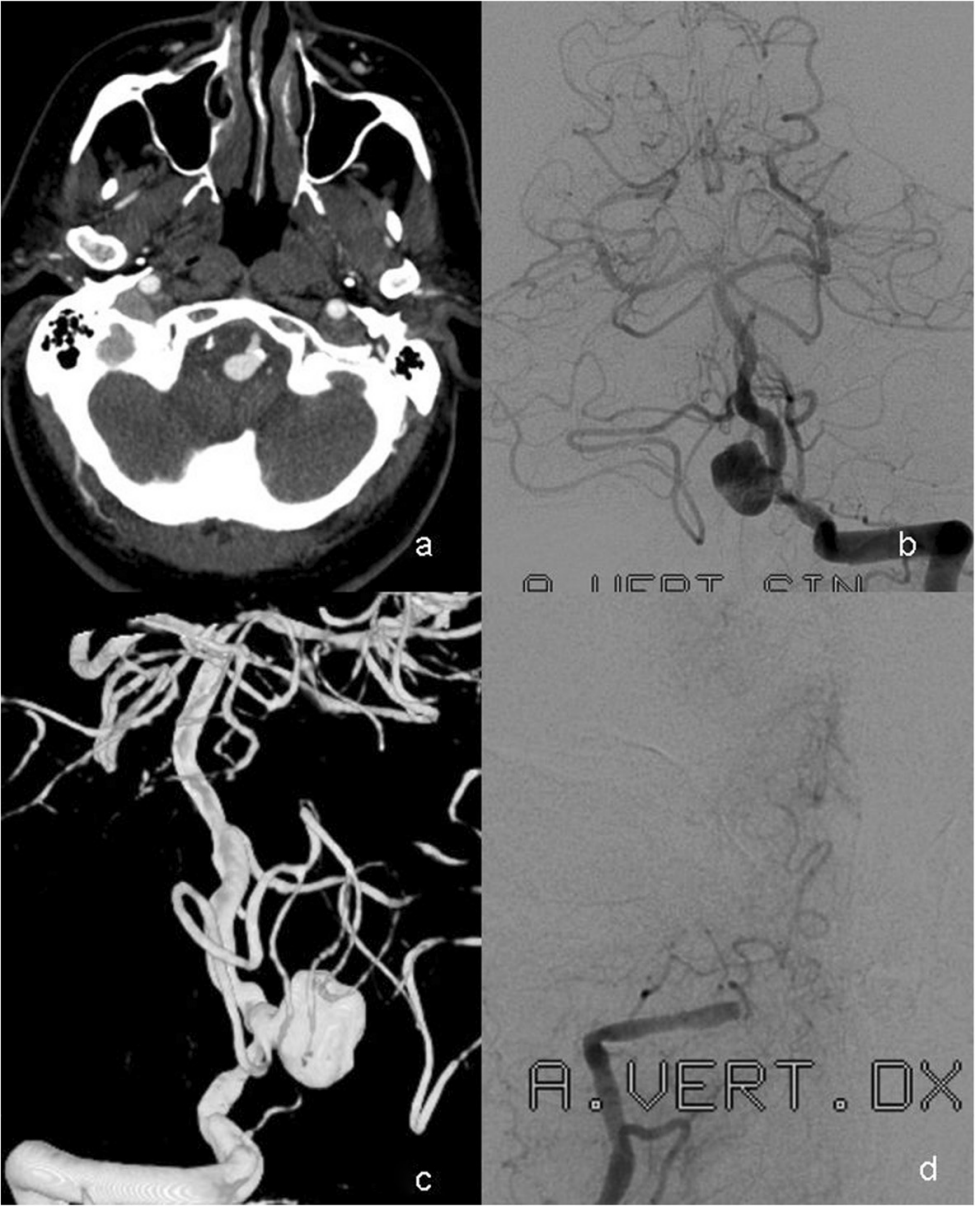
**Preoperative computed tomography and angiographic findings in the posterior fossa. (a)** CT angiography in a 68-year-old male showing the aneurysm with calcified neck imprinted in the brain stem. **(b-c)** Vertebral angiography with contrast injection in the left vertebral artery (AP projection) and rotational angiography with 3D reconstruction of the vertebro-basilar system show an irregularly shaped, saccular aneurysm localized at the left VA-PICA junction. **(d)** Right vertebral angiography shows distal occlusion of the V4 segment.

A 68-year-old Caucasian man was hospitalized because of headache of varying intensity, vomiting, and ataxia of several days’ duration prior to admission. The exact time of symptom onset could not be determined. On admission he was conscious with a Glasgow Coma Scale Score of 14. Non-enhanced computerized tomography showed blood in both occipital horns and a round, hyperdense, partially calcified structure localized just above the foramen magnum and imprinted in the medulla oblongata from the left. Computerized tomographic angiography revealed that the structure corresponded to an irregular aneurysm measuring 10mm×13mm, apparently originating from either the left VA or the left PICA (Figure 1a). Diagnostic angiography showed that the aneurysm originated from the junction left PICA–left VA (Figures 1b, 1c), although the exact anatomical relationship between the aneurysm, PICA and VA remained unclear. The right VA was hypoplastic and occluded (Figure 1d). The lumen of the VA at the origin of the PICA was irregular and highly stenotic, measuring only 1.6mm. The left anterior inferior cerebellar artery was hypoplastic. The left PICA supplied almost the whole left cerebellar hemisphere.

**Figure 2:**
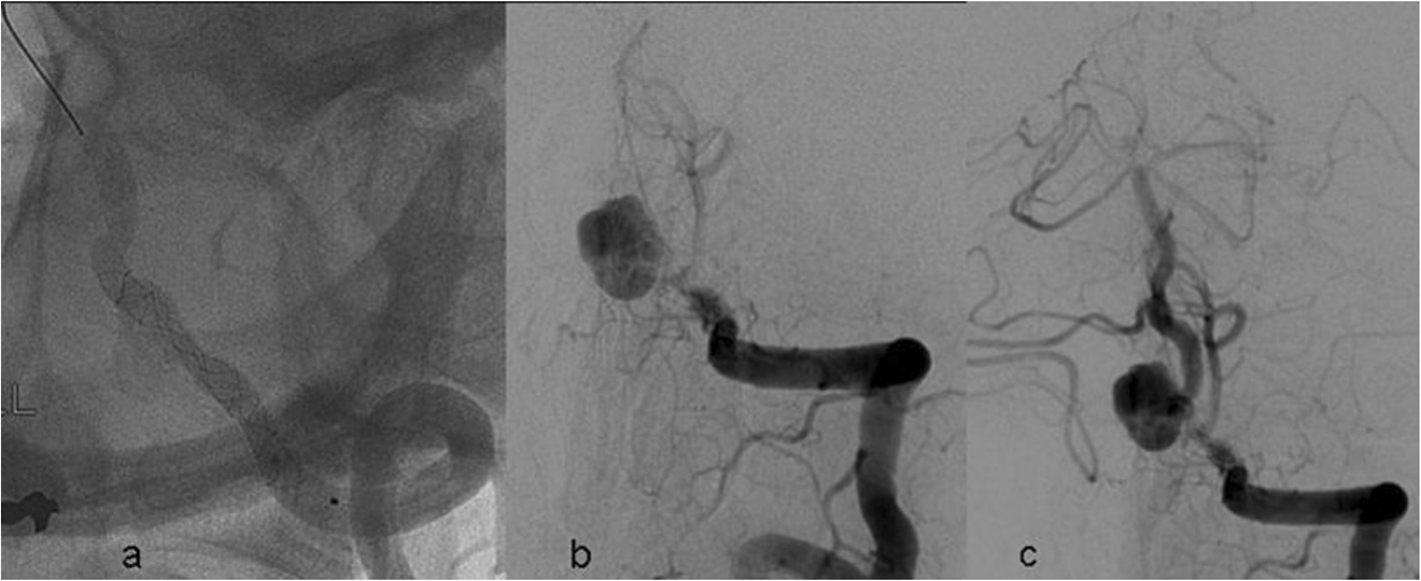
**Treatment of the thromboembolic complication after deployment of the stent. (a)** Unsubtracted left vertebral angiography (left oblique, caudo-cranial view) shows the Silk stent deployed stent deployed in the left V4 segment. **(b)** Thrombo-embolic occlusion of the distal part of the stent. **(c)** Normalized flow in the vertebro-basilar system after local injection of r-TPA and systemic injection of Abciximab.

**Figure 3:**
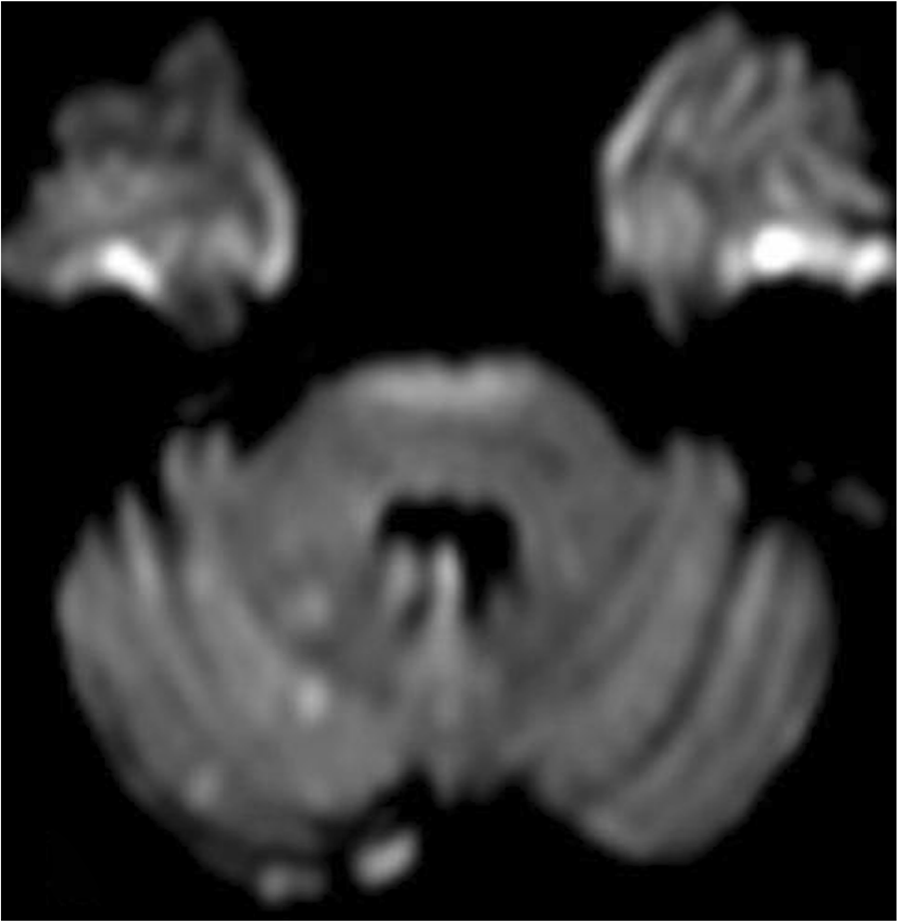
Diffusion-weighted magnetic resonance imaging performed 24 hours after treatment shows several scattered foci of fresh ischemia in the right cerebellar hemisphere.

**Figure 4:**
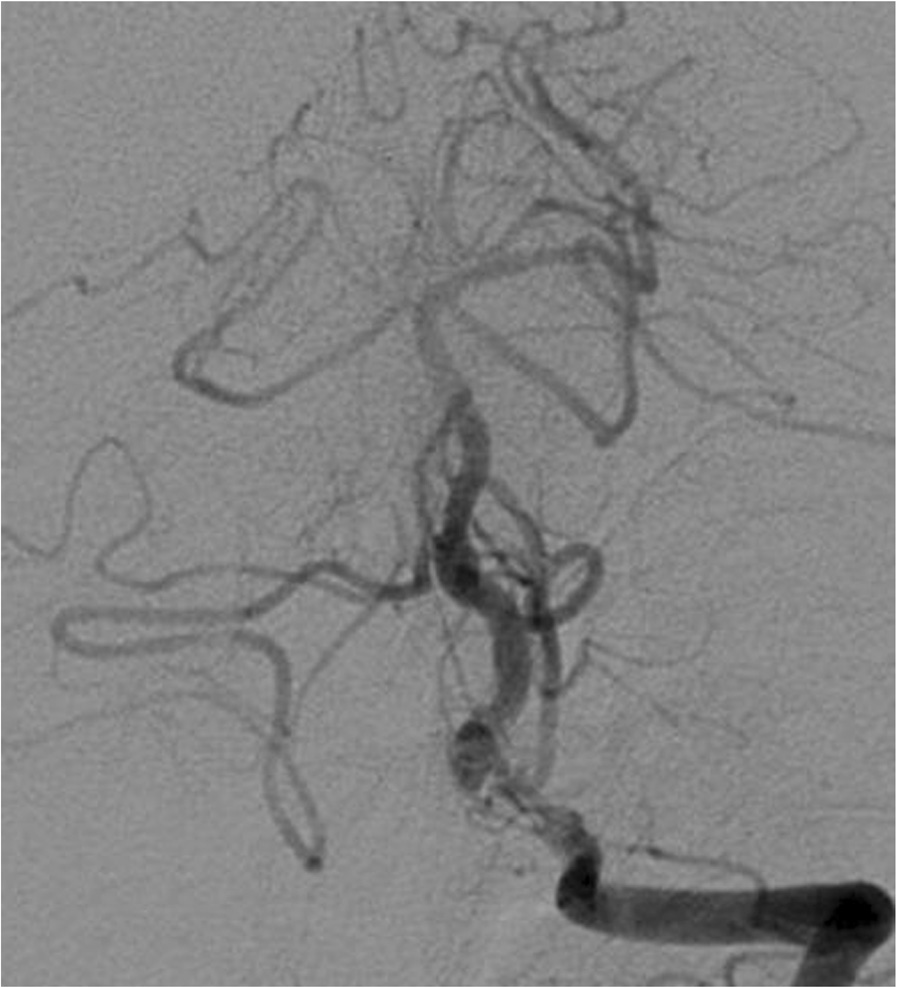
1-year follow-up left vertebral angiography (anteroposterior projection) shows patent left vertebral and basilar artery and significantly reduced lumen of the aneurysm.

**Figure 5:**
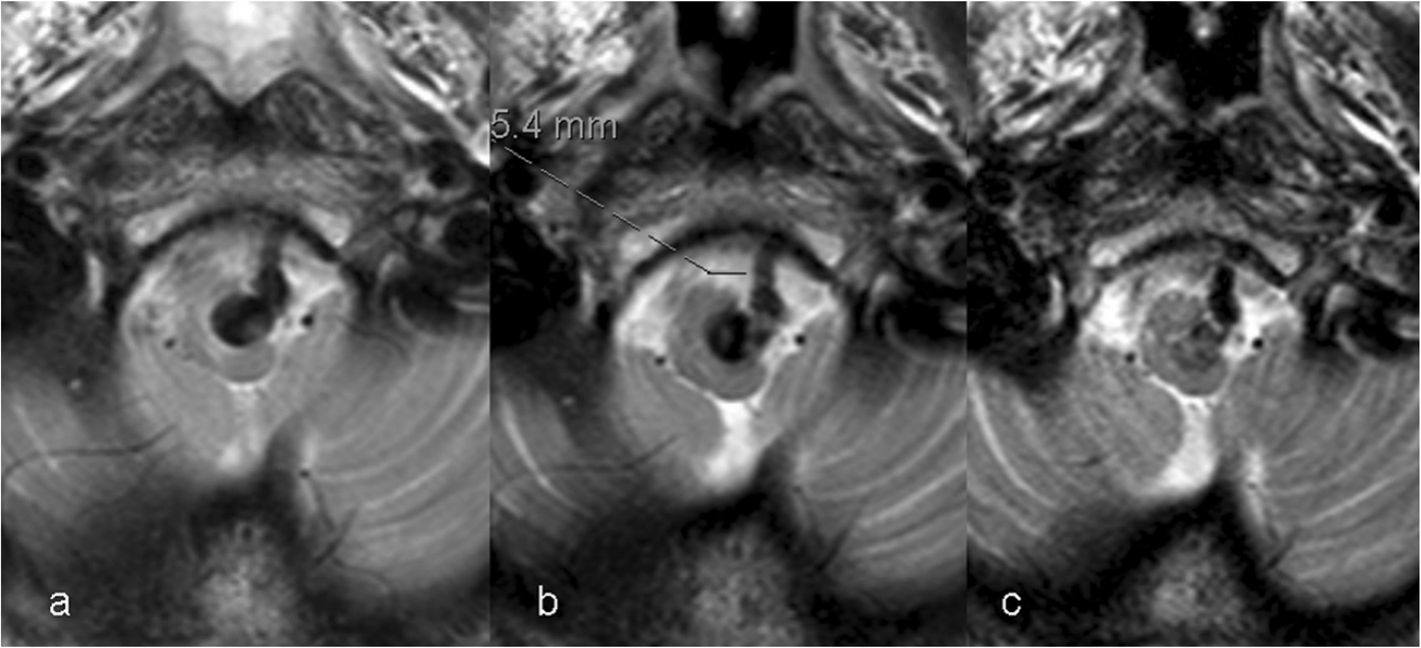
**Gradual shrinkage of the aneurysm during the follow-up period: T2-weighted MR performed (a) prior to treatment, (b) at 4-month follow-up, and (c) 1 year after the treatment show that the aneurysm is diminished in size and the volume of the subarachnoid space around the medulla has been almost completely normalized.**

The patient was under observation and was treated conservatively during the first 9 days after hospitalization. Before the intervention he received a single dose of clopidogrel 600mg. A 6F MPD (Envoy, Cordis, Bridgewater, NJ, USA) guiding catheter was placed in the left VA via a sheath inserted in the right femoral artery. Predilatation of the stenotic left VA was attempted with a compliant 4×10 HyperGlide™ Balloon (eV3/Covidien, Plymouth, MN, USA). In spite of balloon overinflation, the VA stenosis remained unchanged.The balloon was withdrawn, a Vasco 21 microcatheter (Balt Extrusion, Montmorency, France) was navigated through this segment of the left VA, and a 3×25 Silk stent (Balt Extrusion) was deployed in the VA via the microcatheter, covering the origin of the PICA (Figure 2a). Control angiography performed immediately after stent deployment showed contrast stagnation in the aneurysm; however, a control run performed a few minutes later showed thromboembolic occlusion of the basilar artery and the VA distal to the aneurysm (Figure 2b). Intravenous abciximab (ReoPro®, Eli Lilly, Indianapolis, IN, USA) 10mg was administrated immediately, with simultaneous injection of 40mg recombinant thromboplastin activator via the microcatheter into the stent, VA, basilar artery, and both posterior cerebral arteries.Angiography showed reopening of the arteries of the vertebrobasilar system (Figure 2c). Right carotid angiography now showed that the right posterior cerebral artery was fed by the right internal carotid artery (ICA) via a well-developed posterior communicating artery (not shown).Diffusion-weighted magnetic resonance imaging (MRI) performed 24 hours after treatment showed a few minor lesions in both cerebellar hemispheres (Figure 3). The patient recovered slowly but completely. Follow-up angiography performed 4 months and 1 year after endovascular treatment showed reduction of the circulating lumen of the aneurysm and patent arteries of the vertebrobasilar system (Figure 4). Stenosis of the left VA remained stable during follow-up. MRI showed moderate shrinkage of the aneurysm, with gradual reduction of compression on the medulla as well as gradual improvement of anatomical relationships in the foramen magnum (Figures 5a to 5c). At the most recent follow-up, 3.5 years after endovascular treatment, the patient is in good condition and has a normal life.

## Discussion

We present a case of successful endovascular management of a large ruptured aneurysm originating at the junction of the left VA and left PICA by placement of a flow-diverter across the origin of the aneurysm, with gradual complete recovery and shrinkage of the aneurysm on late follow-up imaging.

The endovascular approach offers a therapeutic alternative that is widely accepted and may be preferable in the treatment of some PICA aneurysms when surgical clipping is considered to have an unacceptable risk [1,2]. Encouraging short- and long-term results in most of the cases can be achieved by therapeutic occlusion of the PICA or exclusion of the aneurysm from the circulation with preservation of the PICA [7-9]. Anatomical characteristics of the PICA and VA may restrict the use of balloons and stents in the treatment of aneurysms originating from the VA. Rupture of a PICA aneurysm with local or diffuse spasm of the vertebrobasilar system can complicate the course of endovascular treatment.

The use of stents in the treatment of ruptured aneurysms is generally not recommended because of potential risks of spasm and embolic complications, as well as bleeding, since the use of antiplatelet agents during and after the intervention is mandatory. However, results of recently published studies show that stent-assisted coiling or deployment of stents in parent arteries may offer a promising therapeutic alternative in selected cases, even in the setting of subarachnoidal bleeding [10,11]. Interestingly, several years after the introduction of flow-diverting stents into clinical practice, reports on their use for the treatment of acutely ruptured, predominantly dissecting, and bloodblister-like aneurysms are still relatively sparse [12-14].

Flow-diverting stents redirect blood flow from the aneurysm to the lumen of the parent artery. Such changes of the hemodynamics in the aneurysm and corresponding segment of the parent vessel can lead to spontaneous thrombosis of the lumen of the aneurysm [5,6]. At present, two flow-diverting stents are available, the Pipeline neuroendovascular device (eV3/Covidien) and the Silk stent (Balt Extrusion). The Silk stent is a tubular structure composed of 48 braided nitinol wires with excellent wall opposition and conformability. Flaring at the ends provides apposition in tight curves. Its high-density mesh is designed to redirect the blood flow and induce remodeling of a continuous arterial wall surface. Four helicoidal radio-opaque markers along the full length of the stent allow assessment and control of deployment.

In this particular case, two therapeutic strategies were considered: neurosurgical and endovascular. Surgical clipping was rejected for this patient because of the unacceptable risk of compromising the flow in VA and/or PICA. Endovascular treatment with coils carried almost the same risks, and the authors wanted to avoid permanent compression on the medulla and stenotic VA that could be caused by a huge mass of coils. Additionally, the risk of re-bleeding has significantly decreased in this phase of the disease.

After the meticulous analysis of the clinical picture and imaging studies, and considering the natural course of the disease if not treated, the authors decided to deploy the flow-diverting stent in the left VA. Experience in the treatment of acutely ruptured aneurysms with only flow-diverting stents is limited. The authors decided to treat a ruptured aneurysm using the flow-diverting stent in spite of all known and assumed risks related to the use of this novel remodeling device. In the patient described here, there were three treatment goals: reduce compression on the brainstem, isolate the lumen of the aneurysm from the parent artery, and preserve flow in the PICA and VA. We assumed that flow in the PICA would not be compromised since the radial force of the stent, even if only partially opened, would be sufficient to preserve VA patency. Finally, we assumed that the risks related to surgical clipping or coil occlusion of this aneurysm were much higher than risks related to deployment of the flow-diverting stent in the VA.

## Conclusions

The thromboembolic event that occurred after stent deployment is a well-known complication that sometimes follows stent-assisted aneurysm coiling. Treatment of this complication in the case we describe was quick and effective. The patient’s postoperative course was uncomplicated. Follow-up angiography 4 months and 1 year after treatment, as well as follow-up MRI showed that all goals of the treatment were achieved. Moreover, the stent deployed in the VA preserved its patency and contributed to satisfactory blood flow in the vertebrobasilar system. Stenosis has not shown any sign of progression during the follow-up period. This case indicates that the use of flow-diverting stents in the treatment of some carefully selected ruptured aneurysms could represent a feasible and acceptable therapeutic alternative.

## Consent

Written informed consent was obtained from the patient for publication of this case report and accompanying images. A copy of the written consent is available for review by the Editor-in-Chief of this journal.

## Abbreviations

MRI: Magnetic resonance imaging; PICA: Posterior inferior cerebellar artery; VA: Vertebral artery.

## Competing interests

Financial competing interests:

GG is proctor of Balt Extrusion Company and received reimbursement for conducting the procedure and participation in performing the procedure.

LB, PJ and PAR do not have any financial competing interests.

Non-financial competing interests:

LB, GG, PJ and PAR do not have any non-financial competing interests.

## Authors’ contributions

LB wrote the manuscript. LB, GG, PJ and PAR performed the intervention, participated in the design of the paper and contributed to the editing of the text. All authors read and approved the final manuscript.
